# Accurate modeling of microwave structures in constrained domains using global sensitivity analysis and performance-based pre-screening

**DOI:** 10.1038/s41598-025-27085-8

**Published:** 2025-12-03

**Authors:** Slawomir Koziel, Anna Pietrenko-Dabrowska

**Affiliations:** 1 Engineering Optimization & Modeling Center, Reykjavik University, Reykjavik, 102 Iceland; 2 Faculty of Electronics, Telecommunications and Informatics, Gdansk University of Technology, Gdansk, 80-233 Poland

**Keywords:** Microwave circuits, Simulation-driven design, Behavioral modeling, Experimental validation, Sensitivity analysis, Dimensionality reduction, Pre-screening, Engineering, Mathematics and computing

## Abstract

The significance of behavioral models gradually increases in the design and analysis of microwave components. They are mainly used to replace CPU-heavy full-wave electromagnetic (EM) simulations and to expedite EM-driven procedures, especially optimization. Unfortunately, constructing accurate surrogates is a challenging task. In the case of highly nonlinear frequency characteristics of microwave passives, it is normally feasible only when the structures are parametrized by a small number of parameters belonging to narrow ranges. Design utility of such models is limited. Therefore, we developed a novel methodology for computationally efficient and reliable microwave modeling. The presented approach incorporates dimensionality reduction as well as spatial confinement to lower the cost of training data acquisition and to improve the model predictive power. The former is enabled by rapid global sensitivity analysis, which identifies the directions having major influence on the circuit response variability. These directions span the model domain, which is further confined using the pre-screening mechanism focusing on better-quality designs, as well as the spectral analysis of the selected design subset. The surrogate established in the reduced domain still covers the parameter space parts of primary importance, thereby retaining its design applicability. Excellent accuracy of the proposed technique has been validated through extensive benchmarking against several state-of-the-art methods, whereas design readiness has been demonstrated through circuit optimization under various sets of performance requirements. Physical measurements of fabricated circuit prototypes provide auxiliary yet essential validation of the relevance of the proposed modeling technique.

## Introduction

High-frequency design is reliant on computational tools, with include both circuit and full-wave electromagnetic (EM) simulators^1–4^. The latter are essential to represent cross-coupling effects, substrate-related losses, anisotropy, or the effects of environmental factors. These effect are important for contemporary microwave structures, particularly compact ones^5–7^, multi-layer devices^8^, or metamaterial-based circuits^9^. Reliability of EM-based evaluation comes in pair with high CPU cost, which becomes problematic whenever repetitive analyses are required (parametric optimization^10,11^ tolerance-aware design^12–14^, global or multi-objective design^15–18^. Perhaps the most troublesome is global search, which normally requires thousands of system simulations when using nature-inspired algorithms^19–24^. There have been numerous attempts to expedite EM-driven procedures. Noteworthy techniques include adjoint sensitivities^25^, restricted sensitivity updates^26–28^, parallel computing^29^, mesh deformation^30^, response feature approaches^31^, cognition-driven design^32^, space mapping^33^, response correction methods^34–36^. Other methods include dimensionality and model-order reduction^37–39^. Another class of approaches are surrogate-assisted procedures^14,40–42^, predominantly carried out as machine learning frameworks using metamodels in prediction-correction loops^43–46^. The modeling techniques used in this context include polynomial regression^47^, radial basis functions^48^, kriging interpolation^49^, support vector regression^50^, Gaussian process regression^51^. Other types of models include a large variety of artificial neural networks (e.g.^52–54^, ensemble learning^55^, or polynomial chaos expansion^56^.

The employment of surrogate models may mitigate the issues related to computational overhead of massive EM simulations incurred by, e.g., parametric optimization. Notwithstanding, replacing EM analysis by fast behavioral models in the context of microwave design is rarely an option. Construction of reliable models is severely impeded by the curse of dimensionality^57^, response nonlinearity, and the need for the model to handle broad variations in geometry parameters and frequencies (the latter indispensable for design utility reasons). The methods developed to handle these issues include improved utilization of available data (e.g., ensemble learning^58,59^ or handling training datasets of large sizes. The specific approaches are deep learning^60,61^, or analysis of a particular form of the system outputs (such as orthogonal matching pursuit^62^, high dimensional model representation^63^. Computational effectiveness of the modeling process can be ameliorated by means of multi-fidelity techniques (co-kriging^64^, two-stage Gaussian process regression^65^, or Bayesian model fusion^66^. At this point, it should also be mentioned that computational efficiency of simulation-driven design can also be enhanced by improving the efficacy of the simulation process itself, for example using model-order reduction^93^, adaptive mesh generation^94^, replacing fine-mesh surface modeling with analytical roughness representation^95^, rapid electromagnetic analysis by means of equivalent-circuit abstraction instead of full-wave meshing^96^, or optimizing 3D simulation using goal-oriented meshing and high-order elements^97^. Performance-driven modeling^67–71^ offers yet another possibility to enhance the modeling process by confining it to the regions that only contain high-quality designs^67^. Variable-fidelity^72^ and deep learning versions^73^ have been reported as well. The downside of these techniques is the dependence of the surrogate model domain on the existing performance requirements^68^, which is detrimental to the method versatility.

This article introduces a procedure for efficient and reliable data-driven modeling of microwave structures. The two pillars of the presented approach are dimensionality reduction enabled by fast global sensitivity analysis (FGSA), and volume-wise domain confinement based on stochastic pre-screening. The former allows for spanning the surrogate model domain along a small number of directions that represent most of variations of the circuit outputs, whereas the latter focuses the modeling process on regions containing higher-quality designs. Both mechanisms permit constructing accurate behavioral models using small training datasets. These features have been corroborated using several microstrip components and extensive benchmarking against several state-of-the-art modeling methods. Meanwhile, application case studies (parametric optimization) demonstrate that the reported predictive power improvements of the surrogates do not degrade their design utility. It should be emphasized that, although experimental verification is rarely included in modeling studies, it is essential to demonstrate the suitability of a model for design purposes. Therefore, for supplementary verification, selected designs were prototypes and measured. The measured electrical and field characteristics showed a good agreement with full-wave simulations. This alignment demonstrates the suitability of the developed modeling technique for practical design of microwave components.

This paper brings several original components, which can be summarized as follows: (i) the development of a novel procedure for highly-accurate data-driven modeling of microwave passive components, (ii) the incorporation of domain confinement using dimensionality reduction realized by means of fast global sensitivity analysis, (iii) the incorporation of the extra level of volume-based domain restriction using pre-screening and principal component analysis, (iv) the implementation of the complete modeling framework integrating the mechanisms mentioned, (v) demonstrating its efficacy and superiority over the benchmark, as well as design utility of the proposed surrogates for global optimization of microwave circuits.

## Behavioral modeling by dimensionality reduction and volume-wise domain confinement

This section describes the proposed modeling approach. Formulation of the modeling task (Section “Surrogate modelling of microwave components”) is succeeded by a delineation of the sensitivity analysis procedure (Section “Fast global sensitivity analysis”), dimensionality reduction (Section “Dimensionality reduction”), and volume-wise confinement (Section “Volume reduction”). The complete modeling process is discussed in the Section “Complete modelling procedure”.

### Surrogate modelling of microwave components

**Fig. 1:**
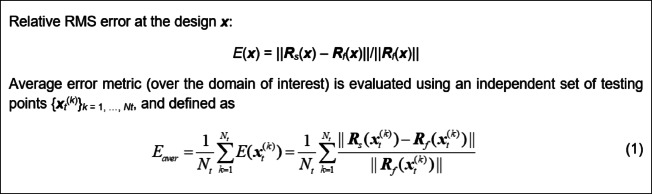
Relative RMS error definition.

We use the notation introduced in Table 1. The purpose of the modeling process is to build a surrogate ***R***_*s*_(***x***) that represents high-fidelity EM-simulated model ***R***_*f*_(***x***) as accurately as possible over the given region of interest and a specified range of frequencies. Here, ***R***_*f*_ stand for the generic response, which, in practice, would be a set of scattering parameters (e.g., *S*_*j*1_, *j* = 1, 2, 3, 4, for a microwave coupler). In this work, the assumed quality metric is a relative RMS error, defined in Fig. 1(see, e.g.^74,75^, for a discussion of alternative metrics). Relative error gives an intuitive account of the model accuracy, which generally agrees with visual assessment of the surrogate- and EM-evaluated system responses. For the model to be suitable for design purposes, the error level within a few percent is typically acceptable.

**Table 1:** Notation used in surrogate modeling of microwave devices.

Symbol	Description	Comments
***x*** = [*x*_1_ … *x*_*n*_]^*T*^	Vector of circuit parameters	Independent circuit dimensions to be tuned in the design process
*X* = [***l u***]	Conventional parameter space	***l*** = [*l*_1_ …, *l*_*n*_]^*T*^ and ***u*** = [*u*_1_ …, *u*_*n*_]^*T*^ are lower and upper bounds on parameters, i.e., we have *l*_*k*_ ≤ *x*_*k*_ ≤ *u*_*k*_ for *k* = 1, …, *n*
***R***_*f*_(***x***)	High-fidelity model (generic response)	Responses of high-fidelity EM simulation model of the circuit at hand. The symbol ***R***_*f*_(***x***) stands for aggregated circuit characteristics evaluated over the frequency range of interest *F*
*S*_*kj*_(***x***,*f*)	High-fidelity model (scattering parameters)	Scattering parameters as functions of the parameter vector ***x*** and frequency *f*; *k* and *j* stand for the circuit ports
***R***_*s*_(***x***)	Surrogate model	Responses of the surrogate model of the circuit of interest

### Fast global sensitivity analysis

The challenges of the modelling process are mainly determined by the number of design variables of the circuit at hand. The modelling error depends on the average value of the distances between the training samples (given other factors, e.g., typical response nonlinearity, equal), which scales poorly in higher-dimensional spaces. Consequently, dimensionality reduction is fundamental for improving the surrogate predictive power and reducing the computational cost of model construction. A possible approach to achieve this is variable screening (the Morris method, Pearson correlation factors, partial correlation coefficients, e.g.^76–78^. Another option is global sensitivity analysis (using Sobol method, Jansen formula, or regression-based methods, e.g.^79–81^. Both aim at eliminating the least significant variables. However, most of the mentioned methods are computationally expensive, typically relying on sets of random samples and perturbation points generated in their respective vicinities. A more serious issue is that leaving out specific parameters may lead to inferior results because circuit responses (especially for miniaturized components) depend on the combined effects of several parameters.

Here, a fast global sensitivity analysis (FGSA) method reported in^82^ is utilized, permitting identification of a set of parameter space directions that are the most influential in terms of their effects on the system response variability. The model domain will be defined using a small subset of such directions (cf. Section “Dimensionality reduction”). These can be arbitrarily oriented, thereby improving the flexibility of the surrogate model arrangement.

The identification of the directions, which affect the component’s responses in the most meaningful manner requires calculating the discrepancies between the responses evaluated at random designs. Subsequently, based on these calculations, a relocation matrix ***S*** is assembled, which is subsequently subjected to spectral decomposition. This process produces eigenvectors ***e***_*j*_​ which represent principal directions of responses’ variation. Their significance is quantified by individual eigenvalues *λ*_*j*_​, *j* = 1,…, *n*. The ultimate domain *X*_*d*_ of reduced dimensionality is spanned by *N*_*d*_ most important eigenvectors. The selection of the number *N*_*d*_ is based on the impact of the directions, which needs to ensure that || [*λ*_1_ … *λ*_*Nd*_]^*T*^ ||/|| [*λ*_1_ … *λ*_*n*_]^*T*^ || ≥ *C*_min_ (in this work, we have *C*_min_ = 0.9, as recommended in^82^. Although the dimensionality of the reduced domain *X*_*d*_ is lower than *n*, the newly identified domain permits representing majority of the component’s response variability. In other words, disregarding the remaining *n* – *N*_*d*_ directions has no negative impact on the design utility of the surrogate model.

### Dimensionality reduction

Constructing the surrogate model domain involves two stages. The first one is dimensionality reduction enabled by FGSA. Using *N*_*d*_ eigenvectors ***e***_*j*_, *j* = 1, …, *N*_*d*_, cf. Section “Fast global sensitivity analysis”, we define.

2 $$X_{d} = \left\{ {{\mathbf{x}} \in X:{\mathbf{x}} = {\mathbf{x}}_{c} + \sum\limits_{{j = 1}}^{{N_{d} }} {a_{j} {\mathbf{e}}_{j} } } \right\} , x_{c} = {\text{ }}\left[ {l + u} \right]/{\text{2}}$$

**Fig. 2:**
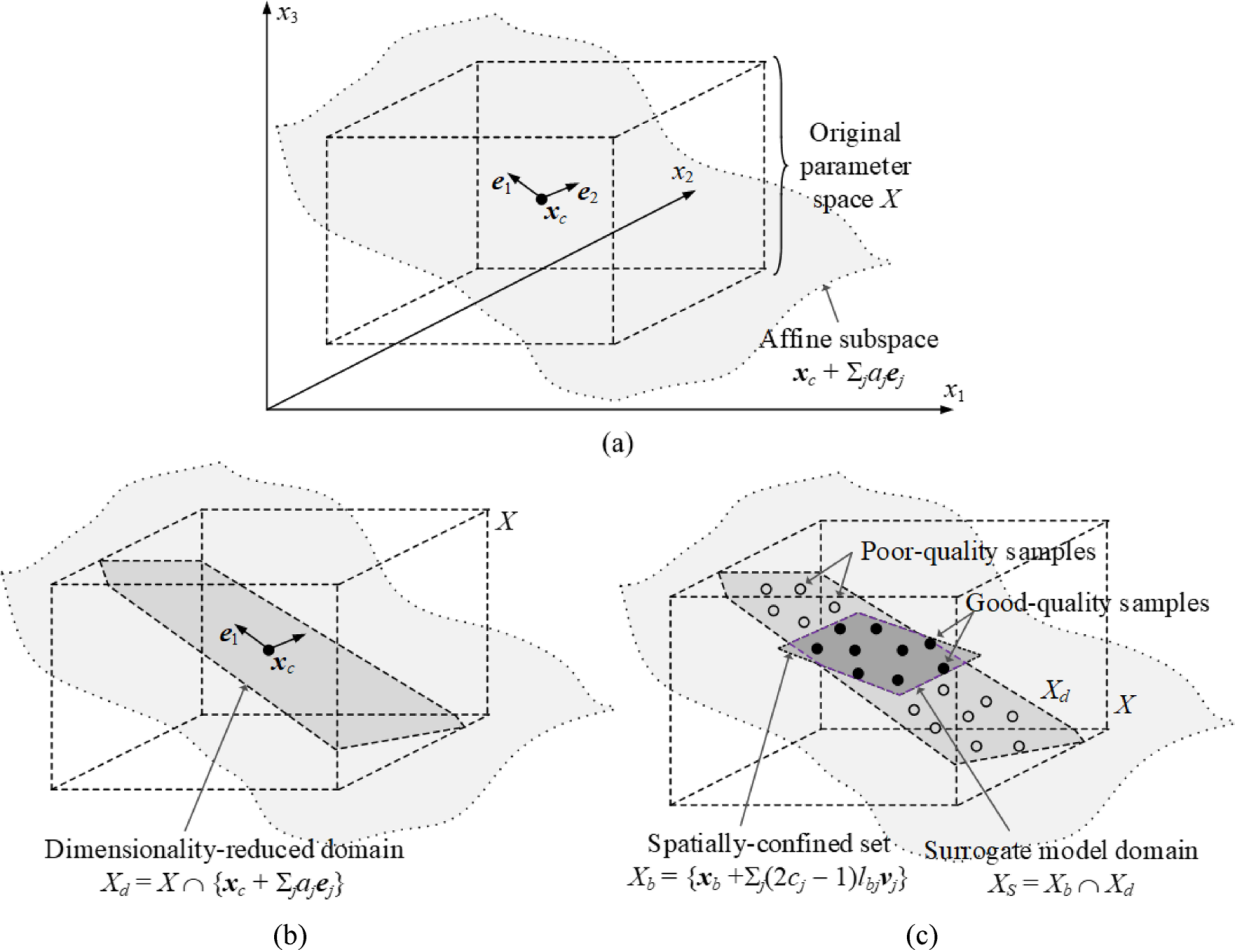
Surrogate model domain *X*_*S*_: dimensionality reduction and spatial confinement: (a) riginal parameter space *X* (here, three-dimensional), and the subspace ***x***_*c*_ + Σ_*j*=1,2_ *a*_*j*_***e***_*j*_ spanned by *N*_*d*_ = 2 eigenvectors ***e***_1_ and ***e***_2_; (b) dimensionality-reduced domain *X*_*d*_, which is an intersection of *X* and the space shown in Fig. 3(a); (c) spatially confined set *X*_*b*_, and the final domain *X*_*S*_ = *X*_*d*_ ∩ *X*_*b*_.

Here, ***x***_*c*_ is the centre of *X*, while the (real) coefficients are denoted as *a*_*j*_, *j* = 1, …, *N*_*d*_. The set *X*_*d*_ has been conceptually illustrated in Fig. 2(a) and (b). Although *N*_*d*_ < *n*, *X*_*d*_ covers the directions associated with the dominant portion of the circuit response variability. Thus, dropping off the remaining dimensions will not compromise design utility of the surrogate constructed therein.

### Volume reduction

Volume reduction is the second stage of model domain construction. It involves quality-based pre-screening. The goal is to identify those parts of the set *X*_*d*_, which contain higher-quality designs. The meaning thereof is problem dependent. For example, for coupling of power dividing circuits, this would mean designs featuring decent impedance matching and port isolation, and/or reasonable levels of power split ratios (e.g., close to zero for equal-power-split circuits).

By *Q*(***x***), we will denote a quality evaluation function, so that *Q*(***x***) = 1 for acceptable designs (e.g., such that |*S*_11_|, |*S*_41_| ≤ − 15 dB at the operating frequency), and *Q*(***x***) = 0 otherwise. By ***x***_*q*_^(*j*)^ ∈ *X*_*d*_, *j* = 1, …, *N*_*q*_, we denote a random set of pre-screening samples (typically, *N*_*q*_ = 50). Finally, by ***x***_*r*_^(*j*)^, *j* = 1, …, *N*_*r*_, we denote a subset of {***x***_*q*_^(*j*)^}_*j*=1,…,*Nq*_, such that *Q*(***x***_*r*_^(*j*)^) = 1 for all *j* ∈ {1, …, *N*_*r*_}. The latter is used to discriminate the region of interest. The analytical confinement procedure works as follows. Given center ***x***_*m*_ = *N*_*r*_^–1^∑_*j* = 1,…,*Nr*_***x***_*r*_^(*j*)^, we perform spectral analysis of the covariance matrix^83^.

3 $${\mathbf{S}}_{r} = \frac{1}{{N_{r} - 1}}\sum\limits_{{j = 1}}^{{N_{r} }} {({\mathbf{x}}_{r}^{{(j)}} - {\mathbf{x}}_{m} )({\mathbf{x}}_{r}^{{(j)}} - {\mathbf{x}}_{m} )^{T} }$$

to yield the eigenvectors ***a***_*k*_, *k* = 1, …, *N*_*d*_. As {***a***_*k*_}_*k* = 1, …, *Nd*_, forms an orthonormal basis within *X*_*d*_, vectors ***x***_*r*_^(*j*)^ have unique expansions

4 $${\mathbf{x}}_{r}^{{(j)}} = \sum\nolimits_{{k = 1}}^{{N_{d} }} {b_{{jk}} {\mathbf{a}}_{k} }$$

It should be noted that Eq. (4) is simply the statement of fact: as the set *X*_*d*_ is *N*_*d*_-dimensional geometrical object, and the eigenvectors ***a***_*k*_ constitute an orthonormal set of vectors spanning the entire *X*_*d*_, any vector ***x*** ∈ *X*_*d*_ can be uniquely expanded as a linear combination of {***a***_*k*_}. Using the expansion coefficients *b*_*jk*_ we define $b_{{j.\max }} = \max \{ k :b_{{kj}} \}$ , $b_{{j.\min }} = \min \{ k :b_{{kj}} \}$ , $b_{{j.0}} = (b_{{j.\min }} + b_{{j.\max }} )/2$ , for *j* = 1, …, *N*_*s*_, as well as ${\mathbf{b}}_{0} = \left[ {b_{{1.0}} \;...\;b_{{N_{d} .0}} } \right]^{T}$ , and ${\mathbf{l}}_{{\mathbf{b}}} = \left[ {l_{{b1}} \;...\;l_{{bN_{d} }} } \right]^{T}$ , where *l*_*bj*_ = (*b*_*j.*max_ – *b*_*j.*min_)/2. Further, we set ***x***_*b*_ **=** ***x***_*m*_ + ***Ab***_0_, where ***A*** = [***a***_1_ … ***a***_*Nd*_]^*T*^ is an *n* × *N*_*d*_ matrix. The smallest *N*_*d*_-dimensional interval containing all vectors ***x***_*r*_^(*j*)^, *j* = 1, …, *N*_*r*_ is

5 $$X_{b} = \left\{ \begin{gathered} {\mathbf{x}} = {\mathbf{x}}_{b} + \sum\nolimits_{{k = 1}}^{{N_{d} }} {(2c_{k} - 1)l_{{b_{k} }} {\mathbf{a}}_{k} } \\ 0 \le c_{k} \le 1,\;\;k = 1,...,N_{d} \\ \end{gathered} \right\}$$

According to this definition, *X*_*b*_ is the region that encloses higher-quality designs according to *Q*(***x***) within the affine subspace ${\mathbf{x}}_{c} + \sum\nolimits_{{j = 1}}^{{N_{d} }} {a_{j} {\mathbf{e}}_{j} }$ .

Finally, the domain *X*_*S*_ of the surrogate model is identified as *X*_*S*_ = *X*_*d*_ ∩ *X*_*b*_ (a visual representation is shown in Fig. 2(c)). The model is rendered in *X*_*S*_ as a kriging interpolative model^84^. The selection of the modelling technique is not the primary concern in this work as our objective is to investigate the potential gains of reducing dimensionality and volume.

The pre-screening procedure can be controlled by appropriate definition of the discrimination function *Q*(***x***), e.g., to intentionally select a specific percentage of {***x***_*q*_^(*j*)^}, or to select the entire set (if all samples are of poor quality). Typically, some of the vectors ***x***_*q*_^(*j*)^ would be of good quality, which enable pre-screening to reduce the overall domain volume, thereby improving the predictive power of the surrogate.

### Complete modelling procedure

**Fig. 3:**
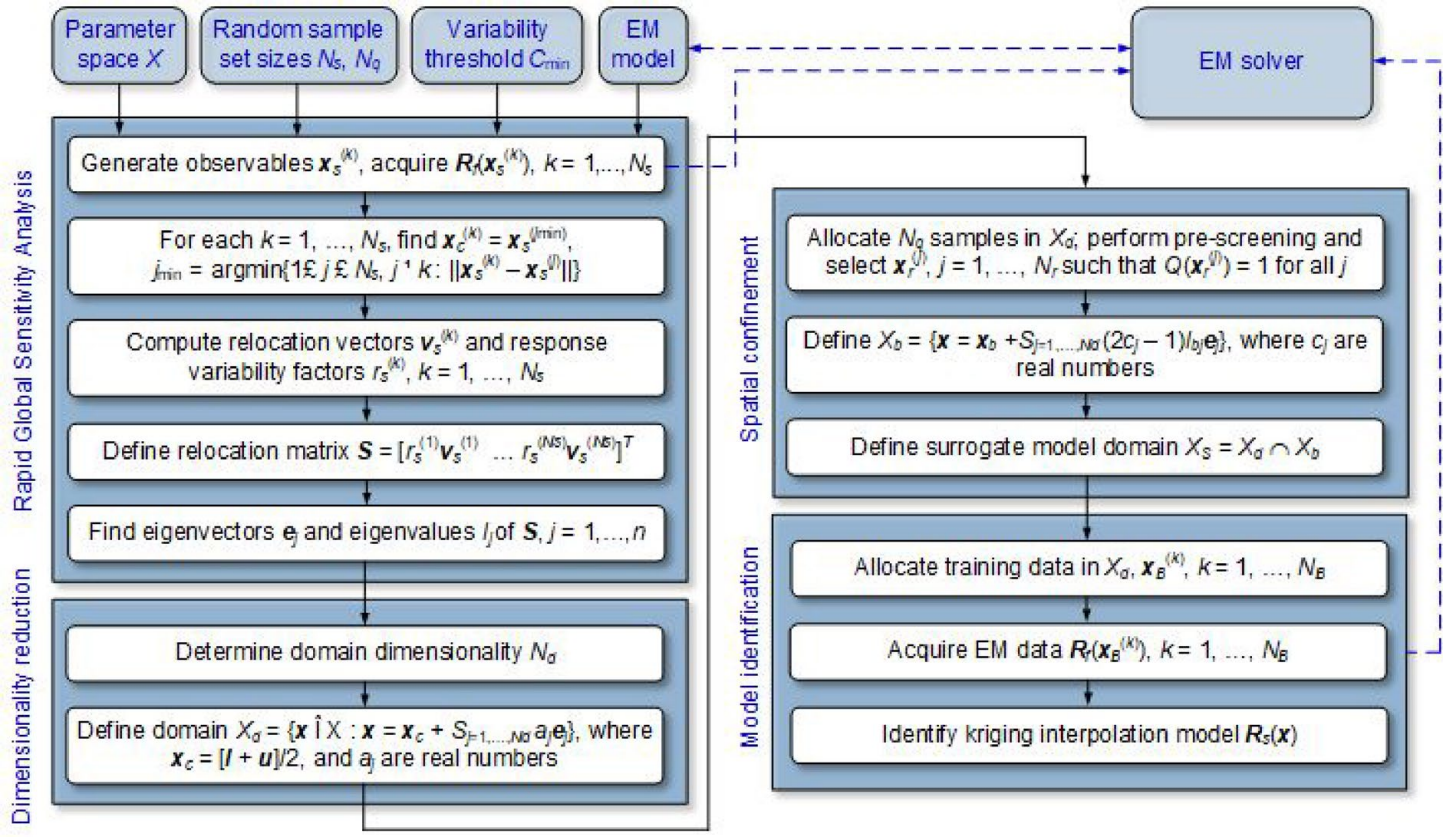
Flowchart of the developed procedure for behavioral modeling of microwave components using dimensionality and volume reduction.

The modelling procedure involves the steps described in the sections “Fast global sensitivity analysis”, “Dimensionality reduction”, “Volume reduction”: sensitivity analysis (Stage I), reduction of the parameter space dimensionality (Stage II) and volume (Stage III), as well as data acquisition and model construction (Stage IV). The flowchart of the procedure is presented in Fig. 3. Note that there are only three control parameters: (i) the threshold *C*_min_ (default value of 0.9), (ii) the number *N*_*s*_ of FGSA samples, and (iii) the number of *N*_*q*_ of pre-screening samples, both set to *N*_*s*_ = *N*_*q*_ = 50.

It should be noted that the modelling procedure proposed in this work differs from previously proposed performance-driven modelling methodologies, such as nested kriging^67^, reference-free modelling^92^, or modelling using dimensionality reduction with global sensitivity analysis^82^. As shown in the next section, our technique incorporates two-stage domain confinement, with automatic determination of the domain size, and is superior to the other performance-driven method regarding predictive power or computational efficiency (or both factors, depending on the specific approach).

## Modeling procedure verification

Here, we discuss numerical verification of the modeling procedure introduced in the section “Behavioral modeling by dimensionality reduction and volume-wise domain confinement”, as well as its benchmarking against several state-of-the-art methods. The test cases include three microstrip circuits with their responses of interest being the scattering parameters versus frequency. In terms of performance figures, we are primarily interested in the predictive power of the surrogates, scalability as a function of the training data set size, and the benefits of incorporating dimensionality and volume reduction. In this section, only the numerical verification of the proposed approach is discussed, whereas the section “Application case studies” provides experimental verification. While it is rarely incorporated in modeling studies reported in the literature, it is crucial to demonstrate the practical suitability of a surrogate model for design purposes.

### Test cases

**Fig. 4:**
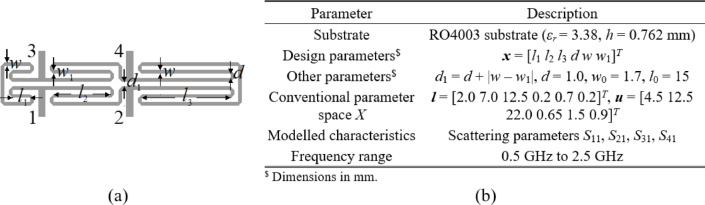
Circuit I: compact rat-race coupler^86^: (a) geometry, (b) parameters pertaining to the modelling process.

**Fig. 5:**
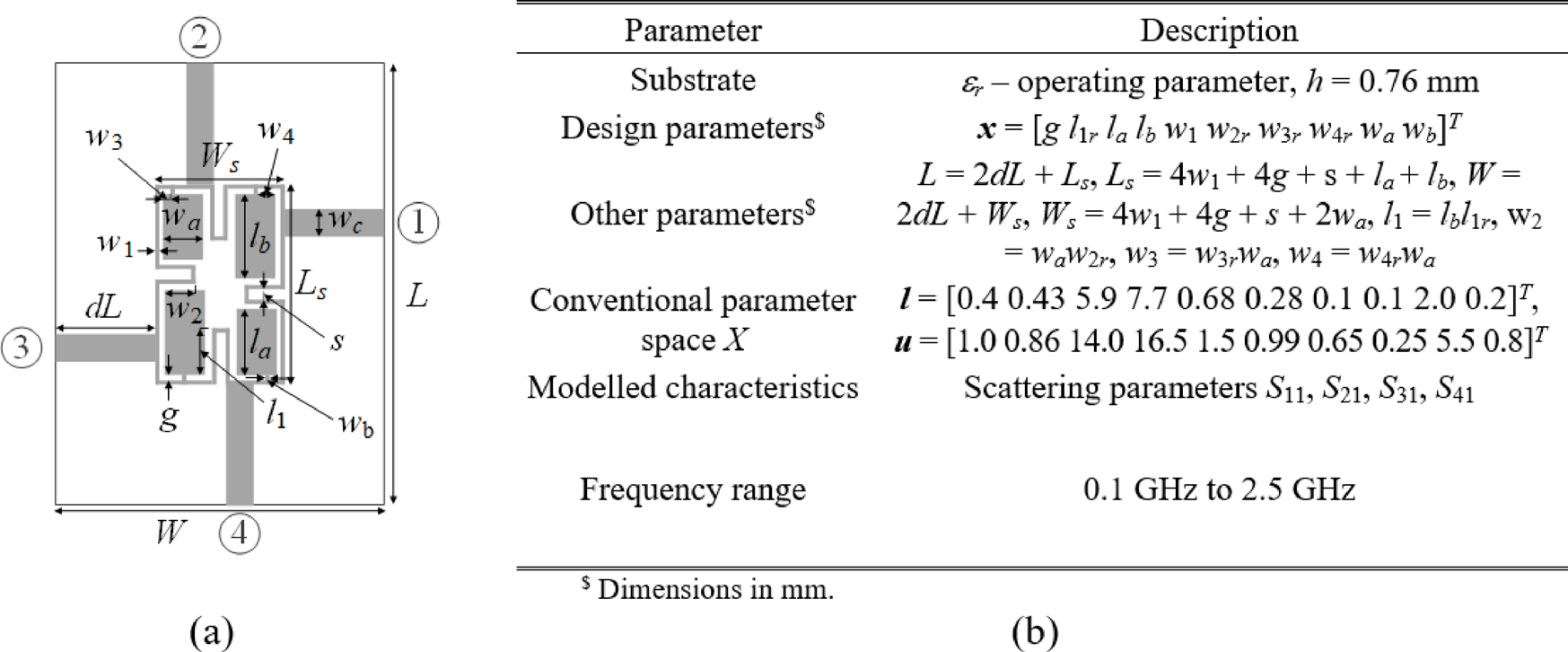
Circuit II: compact branch-line coupler^87^: (a) geometry, (b) parameters pertaining to the modelling process.

**Fig. 6:**
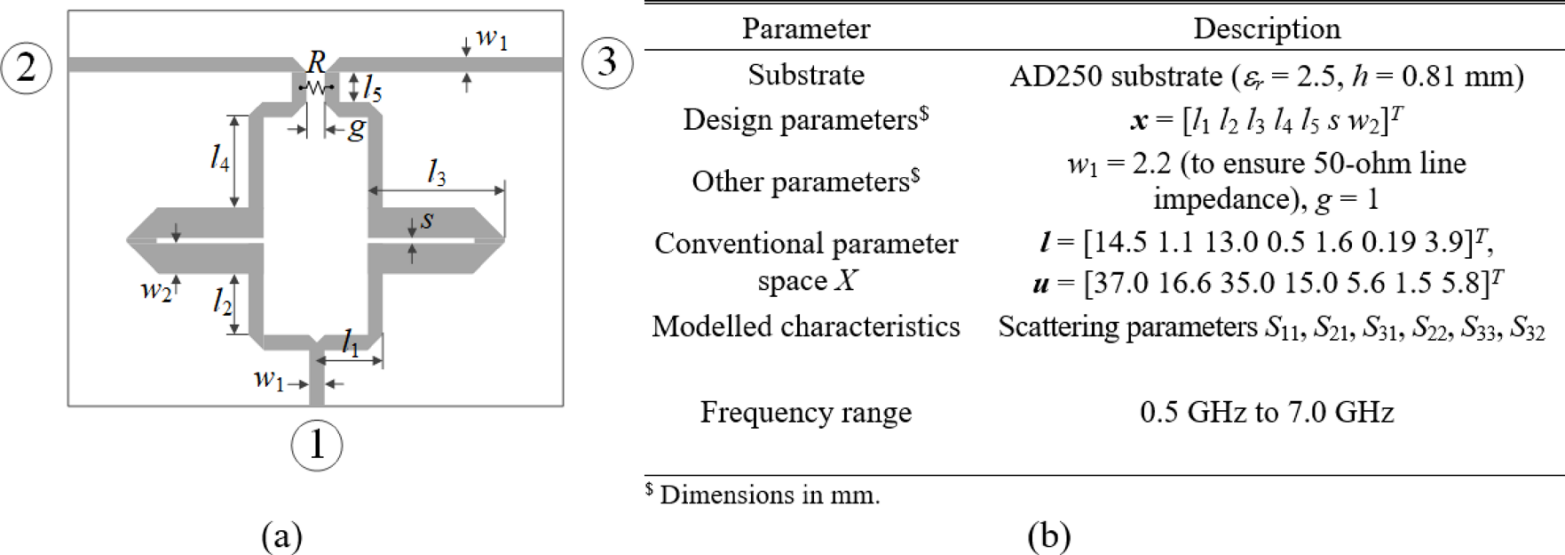
Circuit III: dual-band power divider^88^: (a) geometry, (b) parameters pertaining to the modelling process.

Figures 4 and 5, and 6 show the geometries and essential parameters of the test circuits, labeled Circuit I, II, and III, respectively. The computational models are implemented and simulated in CST Microwave Studio. Challenging nature of the associated modeling tasks should be emphasized, which stems from relatively high dimensionality, broad ranges of geometry parameters (mean ratio of the upper to lower bounds is as high as three for Circuits I and III, and nine for Circuit II), and wide frequency spectra.

### Experimental setup

Numerical experiments carried out here aim at quantifying the potential advantages of dimensionality and volume reduction incorporated into the proposed modelling approach. Towards this end, it is compared to surrogates built in the original parameter space *X*, using a variety of state-of-the-art benchmark methods, performance-driven methods, but also surrogate constructed in a dimensionality-reduced domain (without volume confinement). The details concerning benchmark methods are shown in Table 2. Another objective is to ensure that domain reduction does not impair design utility of the surrogates, which will be verified by the application case studies discussed in the section “Application case studies”.

**Table 2:** Techniques employed for benchmarking the proposed modelling methodology.

Modeling technique	Model setup
Kriging interpolation	Model set up in the full-dimensionality space *X* Gaussian correlation function Second-order polynomial as a trend function
Radial basis functions	Model set up in the full-dimensionality space *X* Gaussian correlation functions Scaling coefficient determined using cross-validation^89^
Artificial neural network (ANN)	Model set up in the full-dimensionality space *X* Feedforward network with two hidden layers Model training: backpropagation
Convolutional neural network (CNN)	Model set up in the full-dimensionality space *X* Uses four filters with filter sizes of [64 128 256 512] Model training: ADAM algorithm^90^
Ensemble learning	Model set up in the full-dimensionality space *X* Uses least-squares boosting with 250 learning cycles Learning rate adjusted through Bayesian optimization^91^
Kriging interpolation	Model set up in the reduced-dimensionality space *X*_*d*_ Domain established using FGSA with the same setup as for the proposed approach (*N*_*s*_ = 50, *C*_min_ = 0.9) Gaussian correlation functions Second-order polynomial as a trend function
Nested kriging^67^	Model constructed in confined domain *X*_*S*_ For this method, the reference design rendition cost must be included in the total expenditure onmodel construction. The number of reference designs *N*_*ref*_ and their acquisition costs are: - *N*_*ref*_ = 12, cost 779 EM simulations (Circuit I) - *N*_*ref*_ = 9, cost 1014 EM simulations (Circuit II) - *N*_*ref*_ = 9, cost 923 EM simulations (Circuit III)
Reference-free performance-driven modeling^92^	Model constructed in confined domain *X*_*S*_ Model domain is determined using pre-screening and orthogonal extension of the inverse regression surrogate The cost of generating observables needs to be included in the overall modeling cost. We have: - *N*_*rnd*_ = 116 EM simulations (Circuit I) - *N*_*rnd*_ = 226 EM simulations (Circuit II) - *N*_*rnd*_ = 78 EM simulations (Circuit III)

As mentioned earlier, the proposed surrogate used the following setup: *N*_*s*_ = 50 samples for FGSA, domain dimensionality *N*_*d*_ established for *C*_min_ = 0.9, and *N*_*q*_ = 50 pre-screening samples. For all test cases, the surrogates are built using the following sizes of training datasets: 50, 100, 200, 400, and 800 samples, with an additional set consisting of 1,600 samples for Circuit III, posing greatest challenge. The design of experiments procedure is Latin Hypercube Sampling^85^. The underlying modelling method is kriging interpolation that employs Gaussian correlation functions and 2nd -order polynomial as a trend function. The predictive power of the models is quantified using average RMS error (cf. Fig. 1).

### Results and discussion

**Fig. 7:**
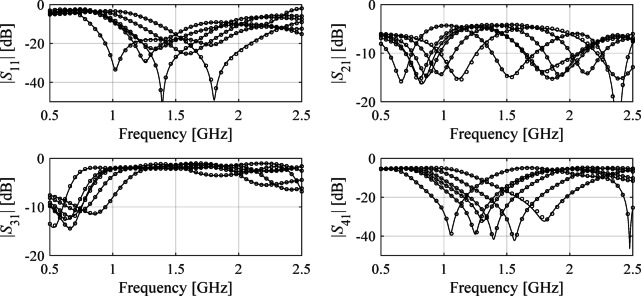
Circuit I: *S*-parameters for specific test designs: EM simulations (—), and the response of the FGSA-based surrogate (o) set up using *N*_*B*_ = 400 data samples.

**Fig. 8:**
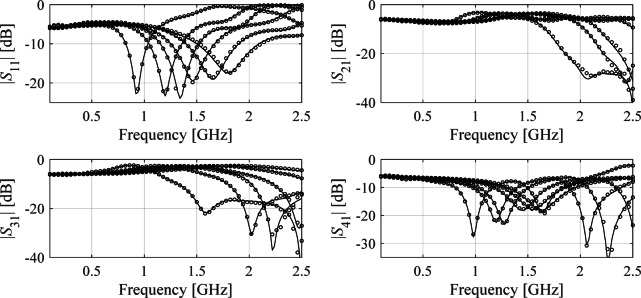
Circuit II: *S*-parameters for specific test designs: EM simulations (—), and the response of the FGSA-based surrogate (o) set up using *N*_*B*_ = 400 data samples.

**Fig. 9:**
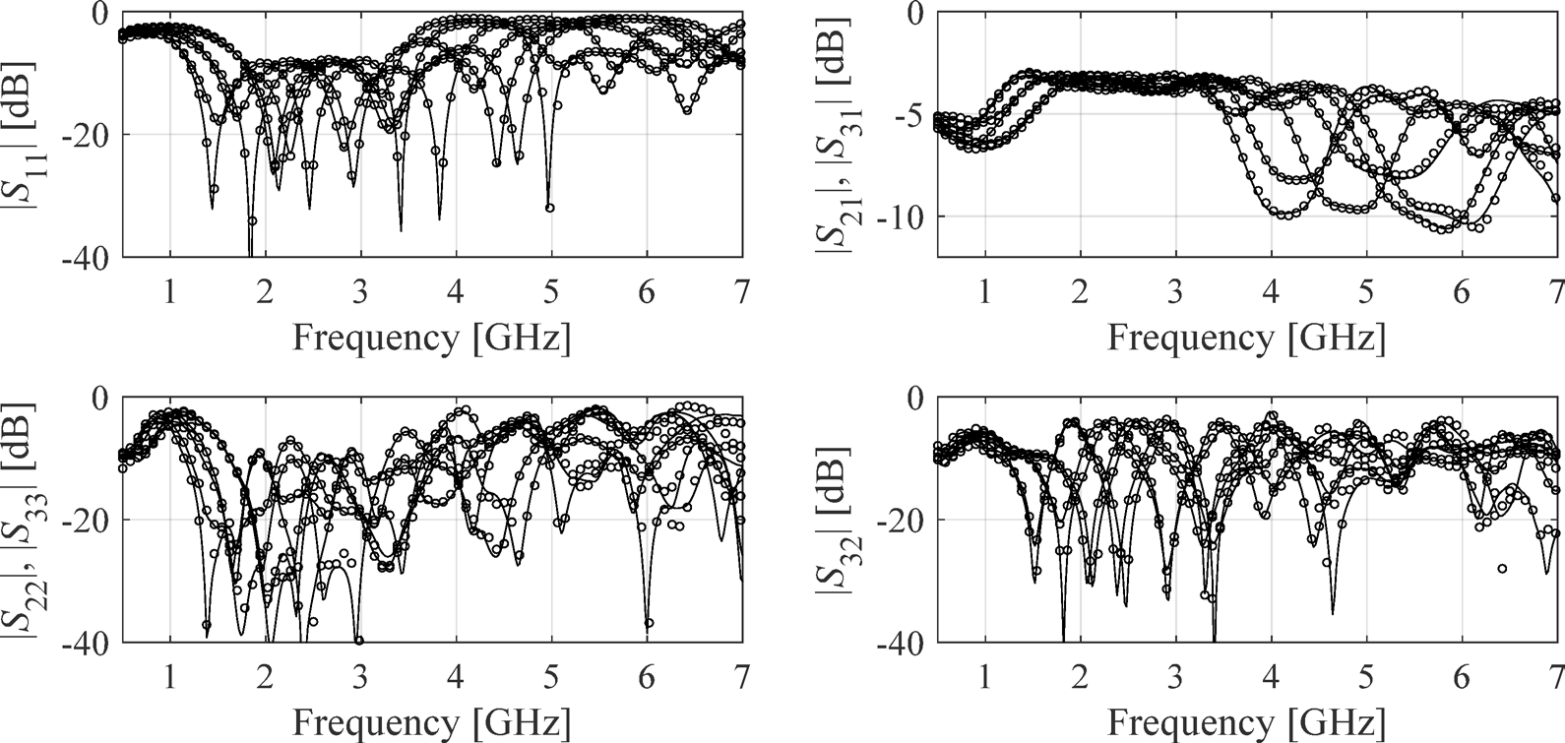
Circuit III: *S*-parameters for specific test designs: EM simulations (—), and the response of the FGSA-based surrogate (o) set up using *N*_*B*_ = 400 data samples.

All circuits considered underwent the sensitivity analysis and the reduced dimensionalities *N*_*d*_ have been assessed as 3, 4, and 4, for Circuit I through III. In^82^, the recommended threshold value *C*_min_ equals 0.9. Here, the selected values of *N*_*d*_ for each circuit correspond to variability factor slightly lower than *C*_min_ (we have 0.89 for Circuit I and 0.8 for Circuits II and III), which is a trade-off assumed to keep the dimensionality as low as possible. The multiplicative reduction factor is around two, three and two for Circuit I, II, and III, respectively. The reduction rate exhibits lowest value for Circuit III, being the most challenging case not only because of the broad range of frequencies but also considerable response nonlinearity over the entire spectrum. Also, in this case, the eigenvalues reduce less rapidly between *λ*_1_ and *λ*_*n*_ (more specifically, from 1.00 to 0.45) as compared to other circuits, which indicates low parameter redundancy. Faster eigenvalue reduction for Circuits I and II is typical for compact structures, both CMRC-based and line- meandering-based. The numerical results are presented in Tables 3 and 4, and 5. Meanwhile, Figs. 7 and 8, and 9 shows the surrogate-predicted and EM-evaluated circuit responses at selected testing locations.

**Table 3:** Modelling results for circuit I.

Modeling method	Number of training samples				
	50	100	200	400	800
Kriging	25.7%	17.9%	13.5%	9.9%	8.0%
RBF	28.3%	19.1%	13.9%	10.3%	8.9%
ANN	18.2%	12.2%	8.0%	7.8%	6.5%
CNN	22.9%	12.7%	8.0%	5.5%	4.5%
Ensemble learning	32.7%	28.1%	25.0%	22.8%	19.1%
Nested kriging^67^	6.9%	5.7%	3.8%	3.5%	3.1%
Reference-free modeling^92^	4.8%	4.2%	3.3%	3.2%	2.6%
Kriging in dimensionality-reduced domain *X*_*d*_^82^	5.9%	3.8%	2.8%	2.4%	1.8%
Kriging in dimensionality-reduced and spatially-confined domain *X*_*S*_ [this work]	4.1%	3.2%	2.4%	2.1%	1.5%

**Table 4:** Modelling results for circuit II.

Modeling method	Number of training samples				
	50	100	200	400	800
Kriging	52.3%	38.3%	31.0%	27.3%	23.3%
RBF	51.8%	40.5%	37.4%	32.8%	27.2%
ANN	29.9%	22.2%	15.2%	10.5%	9.8%
CNN	51.9%	39.9%	30.7%	19.7%	11.5%
Ensemble learning	53.1%	44.4%	41.6%	38.7%	33.3%
Nested kriging^67^	10.0%	7.4%	6.8%	5.1%	4.8%
Reference-free modeling^92^	7.6%	6.2%	4.7%	4.5%	3.4%
Kriging in dimensionality-reduced domain *X*_*d*_^82^	21.5%	15.8%	11.1%	8.5%	6.4%
Kriging in dimensionality-reduced and spatially-confined domain *X*_*S*_ [this work]	14.7%	10.9%	8.3%	7.2%	4.9%

**Table 5:** Modelling results for circuit III.

Modeling method	Number of training samples					
	50	100	200	400	800	1,600
Kriging	63.6%	53.8%	45.2%	40.0%	35.1%	32.3%
RBF	68.9%	55.2%	43.9%	40.8%	37.2%	33.3%
ANN	36.7%	33.2%	24.6%	20.8%	20.3%	19.5%
CNN	89.6%	44.7%	26.0%	17.8%	15.8%	14.2%
Ensemble learning	47.8%	40.6%	38.1%	36.2%	33.6%	30.9%
Nested kriging^67^	32.3%	19.2%	18.1%	15.2%	12.9%	11.2%
Reference-free modeling^92^	23.7%	15.7%	10.8%	7.2%	6.1%	5.9%
Kriging in dimensionality-reduced domain *X*_*d*_^82^	38.9%	28.7%	23.5%	16.6%	12.5%	8.4%
Kriging in dimensionality-reduced and spatially-confined domain *X*_*S*_ [this work]	32.8%	24.2%	15.2%	12.1%	8.5%	6.0%

**Table 6:** Computational cost of model construction.

Circuit	Modeling method	Model building cost^$^					
		*N*_*B*_ = 50	*N*_*B*_ = 100	*N*_*B*_ = 200	*N*_*B*_ = 400	*N*_*B*_ = 800	
I		Kriging	50 [1.7 h]	100 [3.3 h]	200 [6.7 h]	400 [13.3 h]	800 [26.7 h]
		RBF	50 [1.7 h]	100 [3.3 h]	200 [6.7 h]	400 [13.3 h]	800 [26.7 h]
		ANN	50 [1.7 h]	100 [3.3 h]	200 [6.7 h]	400 [13.3 h]	800 [26.7 h]
		CNN	50 [1.7 h]	100 [3.3 h]	200 [6.7 h]	400 [13.3 h]	800 [26.7 h]
		Ensemble learning	50 [1.7 h]	100 [3.3 h]	200 [6.7 h]	400 [13.3 h]	800 [26.7 h]
		Nested kriging	829 [27.6 h]	879 [29.3 h]	979 [32.6 h]	1,179 [39.3 h]	1,579 [52.6 h]
		Reference-free modeling	166 [5.3 h]	216 [7.2 h]	316 [10.5 h]	516 [17.2 h]	916 [30.5 h]
		Kriging in domain *X*_*d*_	100 [3.3 h]	150 [5.0 h]	250 [8.3 h]	450 [15.0 h]	850 [28.3 h]
		Kriging in domain *X*_*S*_ [this work]	150 [5.0 h]	200 [6.7 h]	300 [10.0 h]	500 [16.7 h]	900 [30.0 h]
II		Kriging	50 [2.6 h]	100 [5.3 h]	200 [10.6 h]	400 [21.1 h]	800 [42.2 h]
		RBF	50 [2.6 h]	100 [5.3 h]	200 [10.6 h]	400 [21.1 h]	800 [42.2 h]
		ANN	50 [2.6 h]	100 [5.3 h]	200 [10.6 h]	400 [21.1 h]	800 [42.2 h]
		CNN	50 [2.6 h]	100 [5.3 h]	200 [10.6 h]	400 [21.1 h]	800 [42.2 h]
		Ensemble learning	50 [2.6 h]	100 [5.3 h]	200 [10.6 h]	400 [21.1 h]	800 [42.2 h]
		Nested kriging	1,064 [56.1 h]	1,114 [58.8 h]	1,214 [64.1 h]	1,414 [74.6 h	1,814 [95.8 h]
		Reference-free modeling	276 [14.6 h]	326 [17.2 h]	426 [22.5 h]	626 [33.0 h]	1,026 [51.2 h]
		Kriging in domain *X*_*d*_	100 [5.3 h]	150 [7.9 h]	250 [13.2 h]	450 [23.7 h]	850 [44.9 h]
		Kriging in domain *X*_*S*_ [this work]	150 [7.9 h]	200 [10.6 h]	300 [15.8 h]	500 [26.4 h]	900 [47.5 h]
III	Kriging	50 [4.3 h]	100 [8.6 h]	200 [17.2 h]	400 [34.4 h]	800 [68.9 h]	
	RBF	50 [4.3 h]	100 [8.6 h]	200 [17.2 h]	400 [34.4 h]	800 [68.9 h]	
	ANN	50 [4.3 h]	100 [8.6 h]	200 [17.2 h]	400 [34.4 h]	800 [68.9 h]	
	CNN	50 [4.3 h]	100 [8.6 h]	200 [17.2 h]	400 [34.4 h]	800 [68.9 h]	
	Ensemble learning	50 [4.3 h]	100 [8.6 h]	200 [17.2 h]	400 [34.4 h]	800 [68.9 h]	
	Nested kriging	973 [83.8 h]	1,023 [88.1 h]	1,123 [96.7 h]	1,323 [113.9 h]	1,723 148.4 h]	
	Reference-free modeling	128 [11.0 h]	178 [15.3 h]	278 [23.9 h]	478 [41.2 h]	878 [76.6 h]	
	Kriging in domain *X*_*d*_	100 [8.6 h]	150 [12.9 h]	250 [21.5 h]	450 [38.7 h]	850 [73.2 h]	
	Kriging in domain *X*_*S*_ [this work]	150 [12.9 h]	200 [17.6 h]	300 [25.8 h]	500 [43.1 h]	900 [77.5 h]	

^$^Cost expressed in the number of EM calls. Values in brackets denote the CPU time in hours.

The analysis of results provided in Tables 3 and 4, and 5 reveal that both dimensionality and volume reduction contribute to a significant improvement of the modeling process reliability and computational efficiency. The specific observations have been listed below:

- The RMS errors reported for the surrogates constructed in the full-dimensionality domain are indicative of a challenging nature of the considered test problems. Only for Circuit I, most of the techniques can reach error levels below 10% for the largest training datasets of 400 and 800 samples, which was not possible for Circuit II, let al.one Circuit III, where the typical errors are 20% or more even for the 1,600-sample training set. These accuracy levels render the surrogates unusable for any practical purposes.
- The accuracy improvements achieved with the proposed modeling approach are tremendous. For Circuit I, the error level below 5% has been achieved even for the smallest dataset. For Circuit II, an error of about 10% has been reached with 100 training samples, whereas for the largest set of 800 samples, the error is as low as about 5%. Finally, for Circuit III, an error of about 8% is obtained for 800 sample set, with the level of 6% attained with 1,600 samples. The predictive power of the proposed surrogate is 5.4, 3.8, and 3.7 times better than for the benchmark (averaged over all models in full-dimensionality space) for 800-sample training sets.
- The performance of the proposed modeling methodology regarding accuracy is only matched by other performance-driven methods, including the nested kriging^67^, and reference-free modeling^92^; however, these methods are computationally much more expensive (cf. Table 6). Another drawback is that the specific size of the domain is determined using user-defined control parameters, the value of which must be established by trial and error.
- One of the essential findings is that the contribution of volume reduction to the model accuracy improvement is significant, which can be concluded by comparing the proposed approach with the last benchmark method, i.e., the surrogate constructed in the dimensionality-reduced set *X*_*d*_ (but without volume confinement of the section “Volume reduction”). More specifically, it enables about 1.3-fold reduction of the RMS error when averaged over all considered training data sets and all three circuits. When breaking down to individual datasets, the respective improvement factors are 1.37, 1.3, 1.35, 1.23, and 1.3, for 50, 100, 200, 400, and 800 sets, which means that the improvements are consistent for the training sets of different cardinalities.

From the practical perspective, an important advantage of the proposed methodology is that it permits a rendition of usable models even for relatively small training datasets. In the case of Circuit I, this occurs already for the smallest set of 50 samples, and for the training set of 200 samples in the case of Circuit II. Circuit III is by far the most challenging problem, but even here, the proposed approach yields usable surrogate with the training dataset of 800 samples. At the same time, the models obtained using benchmark methods are grossly inaccurate.

Table 5 reports the computational costs associated with constructing the proposed and the benchmark models. Therein, only the expenses associated with training data acquisition (i.e., EM simulations) have been included because other costs (such as model training) are generally small compared to the overall EM analysis time. The individual simulation times vary depending on the circuit and are as follows: 120 s for Circuit I, 190 s for Circuit II, and 310 s for Circuit III.

## Application case studies

Section 3 demonstrated superior performance of the presented modeling approach in terms of the accuracy of the rendered surrogates as well as computational efficiency of the modeling process. These advantages result from the two major mechanisms incorporated into the procedure: dimensionality reduction and performance-driven volume confinement. From the practical utility standpoint, it is now important to verify whether these enhancements negatively affect the ability of the model to allocate optimal designs for a variety of possible performance requirements. Having in mind the details of the modeling process, the surrogate should ensure sufficient flexibility in this context. Although the domain of reduced dimensionality is reduced to a fraction of the original one (parameter space *X*), it has been defined as accommodating the most relevant directions (i.e., those accounting to most of the circuit response variability) and higher-quality designs (due to the arrangement of the pre-screening process of Sect. 2.4). Notwithstanding, in this section, we put this claim to the test by considering a few application examples, which include parametric optimization of all considered circuits for several design scenarios as described below.

**Fig. 10:**
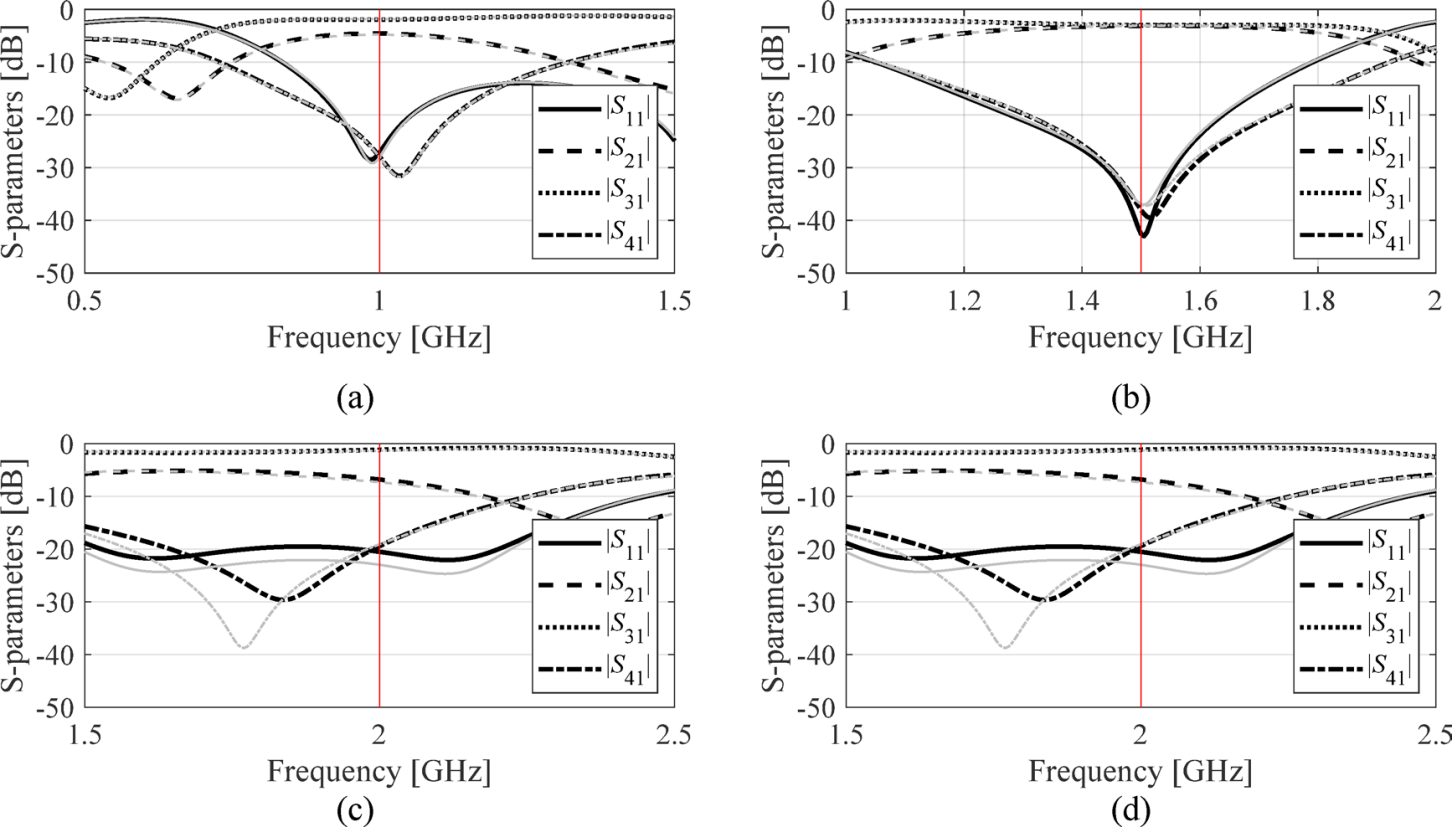
Circuit I: surrogate-predicted (grey) and responses rendered by EM solver (black) both at the optimal design (data set size for surrogate construction comprised four hundred samples). The intended operating frequency *f*_0_ marked using vertical line. The design scenarios are: (a) *f*_0_ = 1.0 GHz, *K*_*P*_ = − 3 dB, (b) *f*_0_ = 1.5 GHz, *K*_*P*_ = 0 dB, (c) *f*_0_ = 2.0 GHz, *K*_*P*_ = − 6 dB, (d) *f*_0_ = 1.2 GHz, *K*_*P*_ = − 3 dB.

**Fig. 11:**
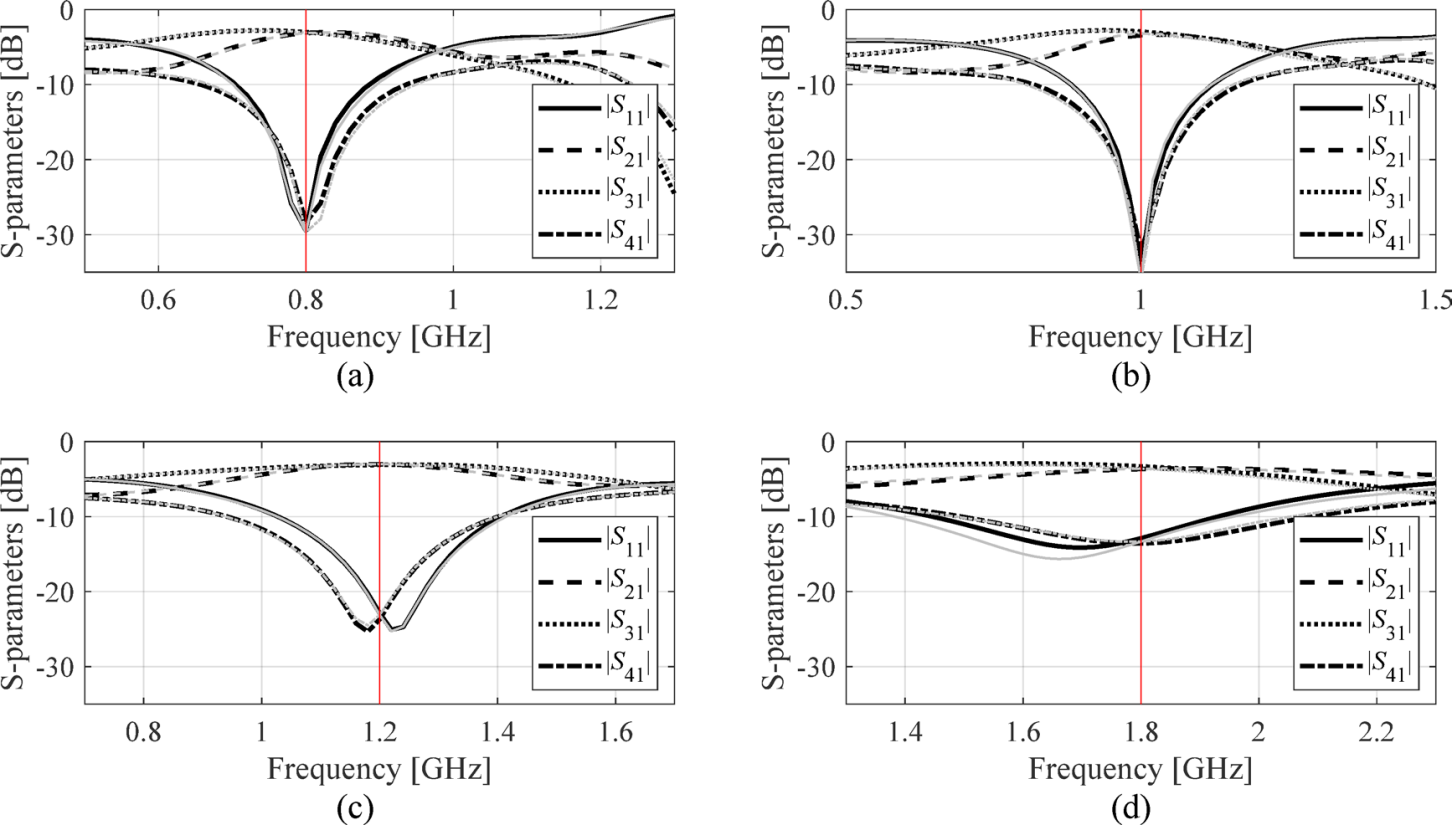
Circuit II: surrogate-predicted (grey) and responses rendered by EM solver (black) both at the optimal design (data set size for surrogate construction comprised eight hundred samples). The intended operating frequency *f*_0_ marked using vertical line. The design scenarios are: (a) *f*_0_ = 0.8 GHz, *ε*_*r*_ = 4.4, (b) *f*_0_ = 1.0 GHz, *ε*_*r*_ = 3.5, (c) *f*_0_ = 1.2 GHz, *ε*_*r*_ = 4.4, (d) *f*_0_ = 1.8 GHz, *ε*_*r*_ = 2.5.

**Fig. 12:**
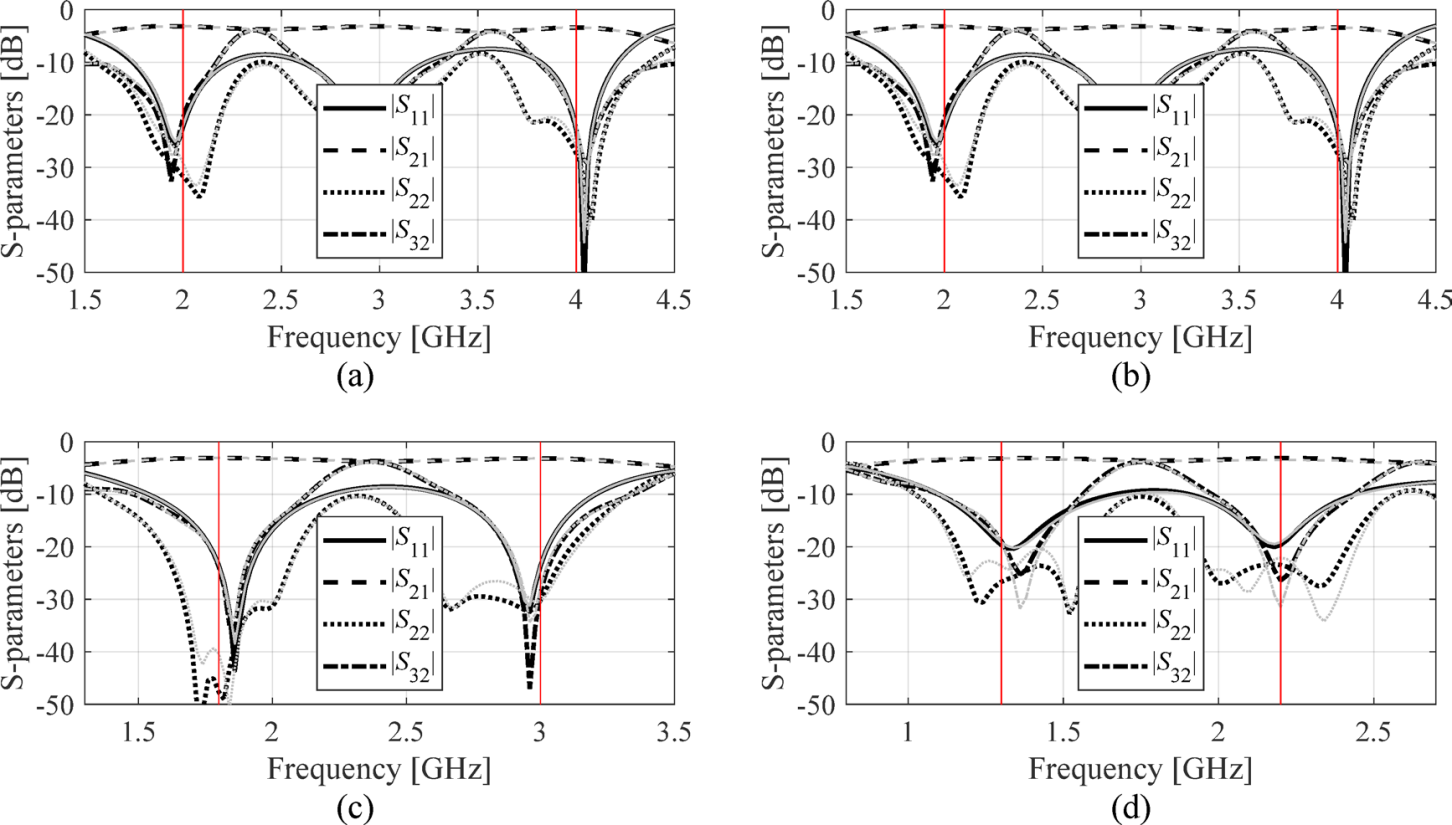
Circuit II: surrogate-predicted (grey) and responses rendered by EM solver (black) both at the optimal design (data set size for surrogate construction comprised eight hundred samples). The intended operating frequencies *f*_1_ and *f*_2_ marked using vertical lines. The design scenarios are: (a) *f*_1_ = 2.0 GHz, *f*_2_ = 4.0 GHz, (b) *f*_1_ = 1.5 GHz, *f*_2_ = 2.45 GHz, (c) *f*_1_ = 1.8 GHz, *f*_2_ = 3.0 GHz, (d) *f*_1_ = 1.3 GHz, *f*_2_ = 2.2 GHz.

The developed surrogate model served to optimize the three circuits for several operating frequencies and power split ratios, and different dielectric substrates (for Circuit II: recall that in this case, relative permittivity *ε*_*r*_ is included in the parameter vector, with values ranging from 2.0 to 5.0). The design objectives have been explained in Table 7. The symmetry of Circuit III automatically satisfies the equal power division requirement; consequently, it is not considered in the optimization process. There are four sets of target operating parameters considered for each circuit. These are center frequency *f*_0_ and power split ratio *K*_*P*_ (Circuit I), *f*_0_ and *ε*_*r*_ (Circuit II), and lower and upper operating frequencies *f*_1_ and *f*_2_, respectively, (Circuit III). The numerical results can be found in Tables 8 and 9, and Table 10. Meanwhile, Figs. 10 and 11, and 12 show the scattering parameters of all circuits at the optimal designs found using the FGSA-based surrogate, as well as corresponding EM simulation data. In all cases, satisfactory designs have been identified, whereas the alignment between surrogate-predicted and EM-evaluated responses is acceptable. This does corroborate design utility of the proposed modeling approach.

**Table 7:** Design application examples for circuits I, II, and III.

Circuit	Design objectives	Other conditions
I	1. Minimize matching |*S*_11_| and isolation |*S*_41_| responses at the target operating frequency *f*_0_; 2. Maintain target power split ratio |*S*_31_| – |*S*_21_| = *K*_*P*_ at *f*_0_.	-
II	1. Minimize matching |*S*_11_| and isolation |*S*_41_| responses at the target operating frequency *f*_0_; 2. Maintain equal power split ratio |*S*_31_| – |*S*_21_| = 0 at *f*_0_;	Circuit implemented on substrate of relative permittivity *ε*_*r*_^$^
III	1. Minimize input matching |*S*_11_| and output matching |*S*_22_| = |*S*_33_| simultaneously at the target operating frequencies *f*_1_ and *f*_2_; 2. Minimize port isolation |*S*_32_| at both *f*_1_ and *f*_2_; 3. Maintain equal power division ratio, i.e., |*S*_21_| = |*S*_31_| at *f*_1_ and *f*_2_.	-

^$^ Relative permittivity is one of the design variables of the modelling process.

**Table 8:** Circuit I: optimization results using the proposed surrogate.

Target operating conditions		Geometry parameter values [mm]					
*f*_0_ [GHz]	*K*_*P*_ [dB]	*l*_1_	*l*_2_	*l*_3_	*d*	*w*	*w*_1_
1.0	–3	3.62	9.7	21.4	0.32	1.48	0.48
1.5	0	2.95	10.7	14.2	0.63	0.86	0.76
2.0	–6	2.50	9.1	12.9	0.20	1.14	0.51
1.2	–3	3.42	9.3	18.7	0.35	1.16	0.44

**Table 9:** Circuit II: optimization results using the proposed surrogate.

Target operating conditions		Geometry parameter values									
*f*_0_ [GHz]	*ε*_*r*_	*g*	*l*_1*r*_	*l*_*a*_	*l*_*b*_	*w*_1_	*w*_2*r*_	*w*_3*r*_	*w*_4*r*_	*w*_*a*_	*w*_*b*_
0.8	4.4	0.55	0.67	12.0	16.3	1.02	0.66	0.37	0.22	4.90	0.34
1.0	3.5	0.79	0.70	9.2	12.5	1.36	0.94	0.49	0.21	4.90	0.38
1.2	4.4	0.99	0.53	9.9	11.0	0.90	0.57	0.36	0.20	2.66	0.54
1.8	2.5	0.81	0.66	6.27	9.4	0.99	0.57	0.38	0.11	3.16	0.65

**Table 10:** Circuit III: optimization results using the proposed surrogate.

Target operating conditions		Geometry parameter values [mm]						
*f*_1_ [GHz]	*f*_2_ [GHz]	*l*_1_	*l*_2_	*l*_3_	*l*_4_	*l*_5_	*s*	*w*_2_
2.0	4.0	26.0	6.78	27.5	4.53	4.79	0.20	5.17
1.5	2.45	30.9	10.1	30.9	8.95	4.36	0.73	4.30
1.8	3.0	26.1	10.2	23.9	10.3	2.60	1.44	4.41
3.3	5.0	31.4	13.3	34.1	10.5	3.35	0.97	3.90

**Fig. 13:**
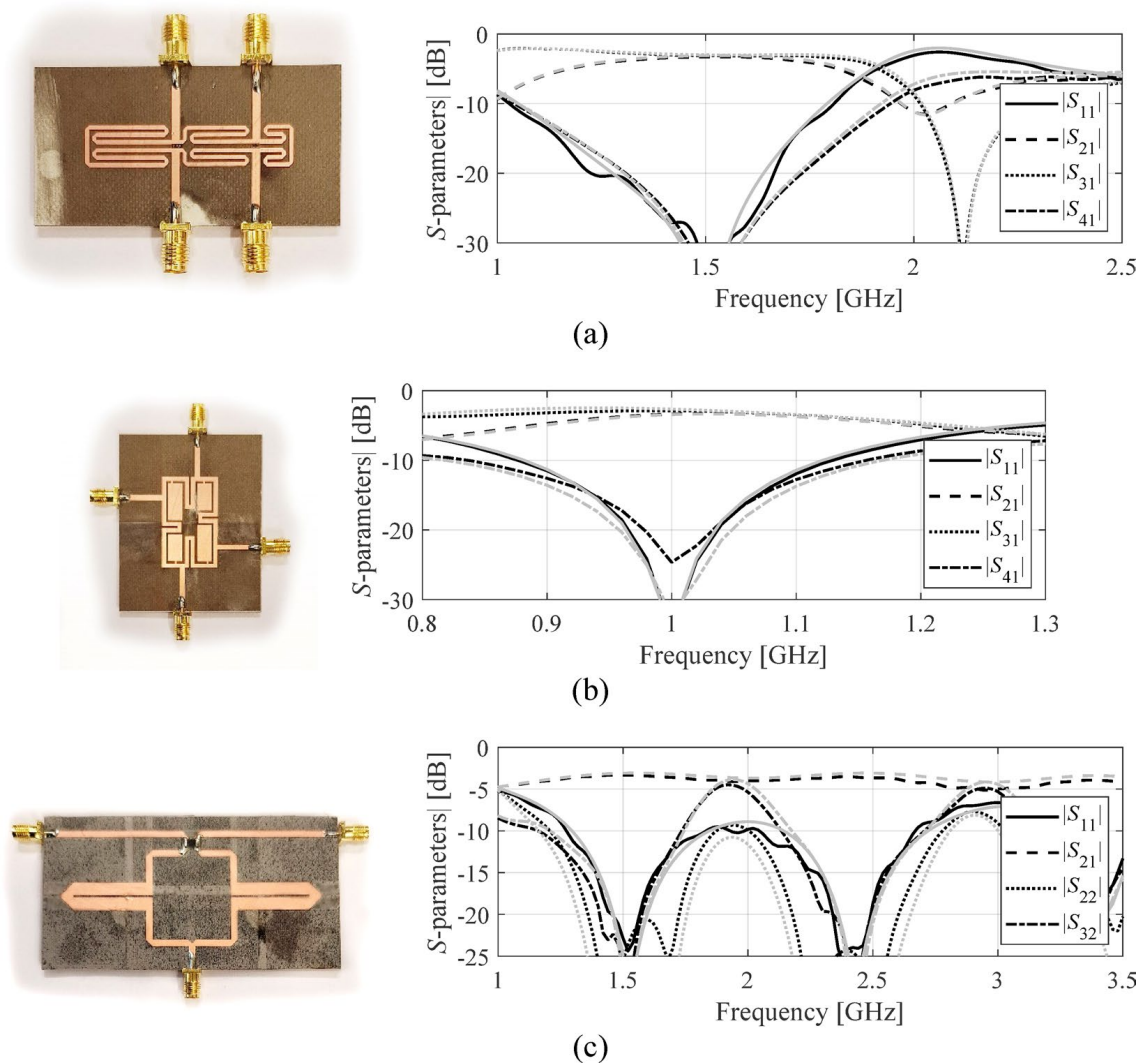
Experimental validation of selected designs optimized using the proposed surrogate model: (a) Circuit I, (b) Circuit II, (c) Circuit III. Gray lines represent EM simulated *S*-parameters, whereas black lines mark the measured characteristics.

As announced in the section “Modeling procedure verification”, the suitability of the proposed surrogate for design purposes is demonstrated through experimental verification of the obtained optimal designs. This allows for a full assessment of the design usefulness of the proposed approach. To accomplish this, selected designs are fabricated and experimentally tested, with measured responses analyzed against full-wave simulation. The designs optimized using the proposed model (the second case for each test circuit) have been fabricated and experimentally verified. Figure 13 presents the circuit prototypes and compares EM-simulated and measured *S*-parameters. Both datasets show satisfactory agreement. Slight differences can be attributed to manufacturing and assembly inaccuracies and the effects of the SMA connectors.

## Conclusion

This article introduced a novel methodology for accurate behavioral modeling of microwave structures. Our approach incorporates two core mechanisms oriented towards the improvement of the model predictive power. The first one is lowering the model domain dimensionality enabled by fast global sensitivity analysis. The second is volume confinement, carried out using pre-screening and spectral analysis of the selected set of higher-quality samples. Constraining the domain allows for reducing the number of training data samples necessary to render an accurate model while retaining its design utility. Ensuring the latter is facilitated by spanning the domain along directions corresponding to the maximum variations of the circuit responses. Our technique has been extensively validated using two microstrip couplers and an equal-split power divider. The considered modeling tasks are challenging with several frequency characteristics to be represented over broad ranges of frequency.

It has been demonstrated that the confinement mechanisms incorporated into the model permit significant improvement of the modeling accuracy when compared to surrogates (kriging, RBF, ANN, CNN, etc.) constructed in the full-dimensionality domain. The levels of the relative RMS errors are from two to seven% for the training dataset consisting of a few hundred samples. At the same time, most benchmark methods exhibit error levels rendering the respective surrogates unusable for any practical purposes.

Design readiness of the proposed models has been confirmed by carrying out several application case studies, i.e., parametric optimization of the considered circuits for different scenarios concerning target operating frequencies, power split factors, and substrate permittivity. The main conclusion drawn from the obtained results is that neither dimensionality nor volume reductions impair design usefulness of our surrogates. Overall, the presented methodology constitutes an attractive alternative to modeling techniques available so far in literature. It is generic and can be combined with a wide range of approximation methods. It is also straightforward to implement, which is important for users with limited background or experience in numerical modeling.

It should also be mentioned that the proposed modeling methodology does not make any specific assumptions regarding the type of microwave circuits to be represented by the surrogates. In other words, it is entirely data-driven, with the training data acquired through full-wave EM analysis. Consequently, it can be, in principle, applied to other types of high-frequency components, such as 3D structures, antennas, or SIW-based circuits. Demonstrating this capability will be a subject of future work.

## Data Availability

The datasets generated during and/or analysed during the current study are available from the corresponding author on reasonable request.
